# Visits to Private Asylums

**Published:** 1904-08-20

**Authors:** 


### VISITS TO PRIVATE ASYLUMS.

By Our Rambling Reporter.

### WEST MALLING PLACE, KENT.

This may be reached in about an hour from London. Malling Station is a mile or so from the Asylum, and Westeringbury between three and four miles. Malling has been known for many centuries. At one time it was a centre of the wool trade, and it is referred to in one of Scott's novels in connection with trials for witchcraft and sorcery. Soon after the Battle of Hastings the Normans pushed on to the neighbourhood and they built the castle known to this day as Gundolph's Keep which now forms a picturesque feature in the landscape, and is so close to the asylum that it seems to form part of the boundary of one of the garden courts where the patients amuse themselves. A thousand years earlier there was a Roman settlement, and remains of villas and baths are still to be found.

The present residence does not go beyond Elizabeth's time, and the central part of the asylum shows unmistakeable evidence of this period, albeit much of the house has been altered and additions made to it. The latter have been well carried out, and there is but little evidence of patchwork. The present proprietor, Dr. Adam, has spent many thousand pounds in bringing the whole mansion and its surroundings up to the modern idea of what is required for the treatment of the insane, and the result must be pronounced successful.

As regards protection from fire, there is, in addition to outside staircases, a very simple arrangement whereby patients could easily get from one room to another if fire broke out in the mansion.

The license dates back about eighty years, and it has been held by Dr. Adam for twenty-one years.

The grounds extend to eight acres and comprise gardens, lawns, avenues, shady walks and orchards. It is fortunate that the grounds are so nice, for Dr. Adam believes strongly in plenty of open-air for his patients, and on the occasion of our visit only one of them was in-doors. Some of the rooms are very good. The patients' drawing-room has scarcely been altered since the Elizabethan period. The old oak panelling is complete and so is the old fire-place. In another room the chimney-piece bears date 1566.

Many of the patients have bedrooms to themselves, and the single-bedded are of large size and well-lighted. They have also special arrangements for being warmed when necessary. All over the mansion there is light and air and cheerfulness. The dining-room is large enough for all the patients to have their meals at the same table, and, of course, one of the higher officials always takes dinner with them.

Patients are admitted from £2 2s. a week and upwards, according to the nature of the case and the accommodation required. We look upon this rate as most moderate.

Dr. Adam's experience in the treatment of mental diseases is hardly second to that of anyone in England, and hence no asylum is more fully and better equipped with what is necessary for the recovery of his patients; and it is situated in a beautiful district within forty miles of London: yet under the existing lunacy laws not more than thirty-nine patients can be resident at one time. It is, practically, always full, or nearly so, which shows that its many good points are appreciated by the people; and one may well ask why patients who are able and willing to pay Dr. Adam's fees should be driven, whether they like it or not, into lunatic hospitals or public asylums, when West Malling Place could be so easily and advantageously enlarged.

